# Effects of a dual-team collaboration model guided by chain management on ECMO initiation and clinical outcomes in critically ill patients: a quasi-experimental study

**DOI:** 10.3389/fmed.2026.1825300

**Published:** 2026-05-05

**Authors:** Yu Ji, Ruiqiang Zheng, Qingjie Zhu, Chaoyu Cao, Qiaoping Chen, Jiangquan Yu, Wei Jiang, Keran Shi, Qiansheng Jin, Fengling Zhang

**Affiliations:** 1 Northern Jiangsu People's Hospital Affiliated to Yangzhou University, Yangzhou, China; 2 Northern Jiangsu People's Hospital, Yangzhou, Jiangsu, China

**Keywords:** chain management, clinical intervention, extracorporeal membrane oxygenation, medical emergency team, rapid response team

## Abstract

**Objectives:**

To develop a chain management–based dual-team collaboration model integrating the rapid response team (RRT) and medical emergency team (MET) and to evaluate its effectiveness in ECMO management in the ICU.

**Methods:**

A chain-based ECMO rescue system was established through literature review, expert consultation, and pilot testing. Fifty-six critically ill patients requiring ECMO were enrolled in a tertiary ICU. Patients admitted from January–December 2023 received conventional MET care (control group, *n* = 28), while those admitted from March 2024–February 2025 received the RRT–MET model (experimental group, *n* = 28). ECMO initiation time, team preparation and circuit priming times, early physiological indicators, complications, and 28-day outcomes were compared.

**Results:**

The experimental group showed significantly shorter ECMO initiation, team preparation, and priming times (all *p* < 0.05). In the VV-ECMO subgroup, oxygenation index improved at 4 h in both groups, and VV-ECMO duration was significantly shorter in the experimental group (*p* < 0.05), but this difference did not remain significant after multivariable adjustment (*p* = 0.23). Complications and 28-day survival favored the experimental group but were not statistically significant. In the VA-ECMO subgroup, blood lactate decreased at 4 h in both groups, and VA-ECMO duration tended to be longer in the experimental group (*p* > 0.05).

**Conclusion:**

The chain management–based RRT–MET model effectively optimized ECMO activation and operational efficiency and was associated with improved process efficiency and showed trends toward improved clinical outcomes, although differences in survival and complications were not statistically significant.

**Implications for clinical practice:**

This structured dual-team pathway enhances coordination, clarifies roles, and reduces delays during ECMO initiation, providing a scalable framework for optimizing emergency ECMO response in critical care settings.

**Clinical trial registration:**

ChiCTR2500103232.

## 1 Introduction

Extracorporeal membrane oxygenation (ECMO) provides temporary cardiopulmonary support for patients with severe cardiac or respiratory failure and has become an essential life-saving technology in critical care settings (1). However, ECMO initiation is highly complex, involving precise coordination of personnel, equipment, and procedures. When combined with the instability of critically ill patients and the high risk of complications, inefficiencies in ECMO preparation and activation may directly compromise treatment effectiveness and patient prognosis (2). Issues associated with conventional emergency treatment include unclear divisions of labor, ambiguous responsibilities, and inefficient team collaboration. These problems increase the risks of delays, potentially leading to deterioration in the patient’s condition, thereby seriously impairing treatment outcomes and prognosis (3–5). To address these challenges, multidisciplinary team (MDT) approaches and rapid response team (RRT) systems have been increasingly incorporated into ECMO-related emergencies, improving the timeliness of rescue and the success rate of critical interventions (6, 7). However, current models focus on emergency responses during the ECMO initiation phase, neglecting the full treatment and management processes after ECMO establishment (8).

Currently, in-hospital emergency care for critically ill patients relies primarily on two models, namely, the medical emergency team (MET) and the RRT (9). The MET is a specialized team established to reduce both in-hospital mortality and the incidence of adverse events. It can respond rapidly to emergencies and deliver emergency treatment, thus enhancing the success of rescue (10, 11). In contrast, the function of the RRT involves rapid responses to emergencies. The RRT performs early assessments and interventions when the condition of a patient outside the intensive care unit (ICU) deteriorates, thereby either reducing unnecessary admission to the ICU or facilitating the timely transfer of the patient to the ICU for standardized treatment (12, 13). Although the value of both models has been verified, their application in ECMO treatment is poorly documented. Recent studies underscore the importance of establishing a coordinated, mobile, interdisciplinary ECMO team to optimize outcomes (14). Chain management-originally derived from supply chain management-focuses on strengthening the continuity, coordination, and efficiency of each operational link within a process (15, 16). This concept aligns closely with the sequential, interdependent nature of ECMO care, which typically follows the continuum of “activation-transport-cannulation-monitoring-weaning.”

Against this background, the present study evaluates the effects of a dual-team collaboration model—integrating RRT and MET—guided by chain management principles. By examining its impact on ECMO initiation efficiency, physiological indicators, ECMO duration, complication rates, and 28-day outcomes, this study aims to provide evidence for optimizing ECMO workflows and enhancing the effectiveness of interdisciplinary team collaboration in critical care settings. Given the observational and quasi-experimental design of this study, causal inferences are inherently limited, and the findings should be interpreted as hypothesis-generating.

## 2 Methods

### 2.1 Study design and setting

This study was a single-center, two-arm, parallel-group quasi-experimental study evaluating the effects of a dual-team collaboration model guided by chain management on ECMO initiation and clinical outcomes in critically ill patients. The study was conducted in the General Intensive Care Unit of a tertiary Grade A hospital in Jiangsu Province, China, from January 2023 to February 2025. Ethical approval for this study was obtained from the Institutional Ethics Committee in 2024 (Approval No. 2024ky134), and all participants provided written informed consent prior to enrolment.

### 2.2 Participants

Participants were recruited between January 2023 and February 2025 from the General Intensive Care Unit of a tertiary Grade A hospital in Jiangsu Province. Eligible patients were those who: (1) provided written informed consent; (2) were aged 18 years or older; (3) met the clinical indications for ECMO and received ECMO treatment; and (4) were admitted to the Department of Critical Care Medicine. Patients were excluded if they: (1) had vascular conditions preventing cannulation; (2) received ECMO without subsequent transfer to the ICU; or (3) had an ECMO runtime of less than 4 h.

The sample size was calculated using the formula for comparing two independent means, assuming *α* = 0.05, *β* = 0.10, and an effect size of *δ*/*σ* = 0.9, yielding a minimum requirement of 26 participants per group. Allowing for a 5% attrition rate, 28 patients were included in each group, resulting in a final sample of 56. Patients managed under the conventional MET-led ECMO workflow between January and December 2023 formed the control group, whereas those treated with the chain management–guided RRT–MET model between March 2024 and February 2025 comprised the experimental group.

A non-concurrent design was chosen to prevent cross-contamination between the two models, as simultaneous implementation could have led to diffusion of the RRT-MET protocol into the control group. To minimize temporal bias, both groups underwent identical standardized training immediately before their respective study periods, and no hospital-wide ECMO protocol or equipment changes occurred between 2023 and 2025. Moreover, the core ICU physician and nursing teams remained stable throughout the study period, with no major staff turnover or restructuring. ECMO decision-making criteria, weaning protocols, and peri-ECMO management standards were also unchanged between the two time periods.

### 2.3 Study procedures

First, a standardized face-to-face training program was simultaneously implemented for members of the dual-team collaboration group and the conventional care group. The 4-h training included: (1) an introductory lecture delivered by the principal investigator on the chain-management-guided dual-team collaboration model, including ECMO initiation workflow, role delineation, communication pathways, and emergency response algorithms; and (2) a practical workshop led by the ECMO specialist on standardized assessment, catheterization preparation, device initiation, and checklist-based safety verification. All participating staff were required to complete a qualification test after training, with a passing score of ≥80/100. The quasi-experimental study was then initiated. Research nurses screened critically ill adult patients requiring ECMO initiation based on predefined criteria.

Patients admitted during the first study phase were assigned to the control group receiving standard ECMO initiation procedures, whereas those admitted during the subsequent phase were assigned to the intervention group managed using the chain-management-guided dual-team collaboration model. Baseline clinical data were collected before ECMO initiation. The intervention involved structured collaboration between the critical care team and the ECMO specialist team, including rapid activation of the ECMO initiation chain, parallel task execution, and real-time closed-loop communication, until completion of ECMO cannulation and stabilization. All clinical procedures followed international ECMO guidelines. Outcome assessments—including initiation time, complication rate, and early clinical outcomes—were conducted by independent assessors who were blinded to group assignment. Data collection continued until ECMO stabilization or discontinuation according to clinical decisions.

### 2.4 Control group

Participants in the control group received routine ECMO emergency care delivered by the Medical Emergency Team (MET). All members of the ECMO MET team had ≥5 years of experience in critical care, completed standardized in-hospital ECMO technical training, and obtained qualification certification. Patients received conventional ECMO nursing care provided by the MET team, including: (1) assisting physicians in circuit preparation, priming, and connection following standardized procedures; (2) continuous monitoring of vital signs and maintenance of airway patency; (3) close observation for early detection and management of complications; (4) implementation of effective sedation and analgesia; (5) anticoagulation monitoring and adjustment according to protocols; (6) provision of progressive nutritional support; and (7) coordination of nursing care throughout ECMO maintenance and weaning.

### 2.5 Experimental group

#### 2.5.1 Development of the dual-team collaboration model guided by chain management

The dual-team collaboration model was developed through a structured, evidence-informed process. First, an eight-member research team-comprising the department director, ICU physicians, and senior ECMO nurses—conducted a systematic literature search across major Chinese and international databases following standardized evidence retrieval procedures. Thirteen high-level studies were ultimately included, covering expert consensuses, evidence summaries, and observational research related to ECMO management, rapid response teams, and chain management. Based on this evidence and existing clinical practice, the team drafted an initial framework integrating the RRT and the MET within a chain management pathway, defining team composition, competency requirements, material preparation, and standardized procedures before, during, and after ECMO initiation.

**Figure 1:**
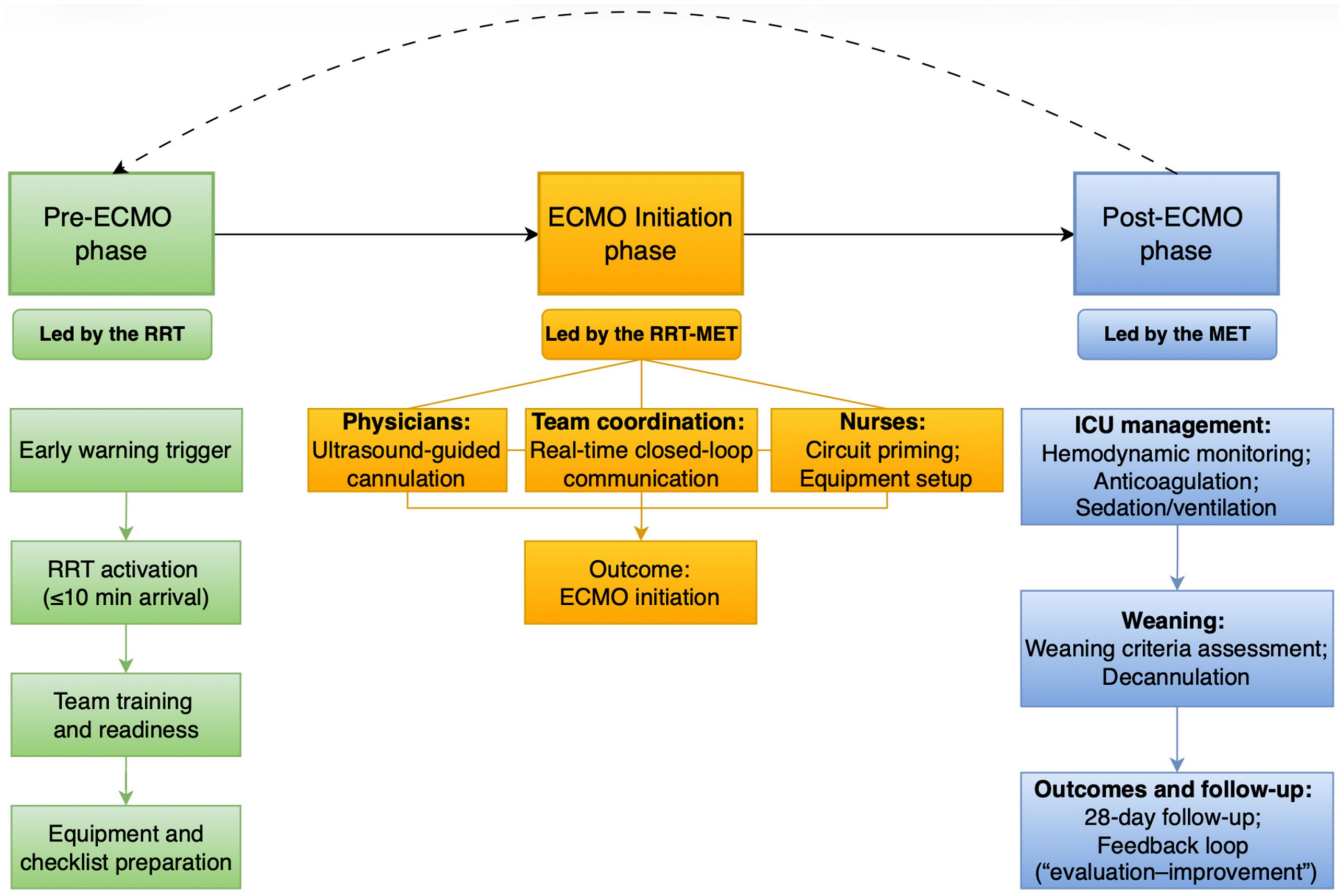
Chain management–guided RRT–MET collaborative model for ECMO care across pre-ECMO, initiation, and post-ECMO phases. RRT, rapid response team; MET, medical emergency team; ECMO, extracorporeal membrane oxygenation; Solid arrows indicate workflow progression; Parallel arrows indicate simultaneous task execution; Dashed arrows indicate feedback and quality improvement processes.

Subsequently, an expert panel of 10 specialists in critical care and cardiovascular surgery participated in consensus meetings to refine the preliminary framework. Using a structured discussion and revision process, expert feedback was incorporated to produce Version 1.0 of the model. To ensure feasibility and operability, high-fidelity simulation drills and a small pilot test were conducted. Issues such as role coordination and emergency material response were identified and optimized, leading to the finalized dual-team collaboration model guided by chain management. This model established a full-process ECMO emergency care chain to promote timely initiation, standardized management, and multidisciplinary coordination (Figure 1).

#### 2.5.2 Description of the dual-team collaboration model guided by chain management

The experimental group received ECMO care under the dual-team collaboration model guided by chain management. The model was jointly led by the departmental director and head nurse, integrating a RRT and a MET. The RRT consisted of five subgroups staffed by ICU chief physicians and senior nurses, all of whom had completed provincial-level ECMO technical training and certification. Following an eight-week program of theoretical training, skills practice, and transfer simulation drills—and quarterly refresher training thereafter—a feed-forward preparation chain of “reservation–training–assessment” was established to ensure team readiness. When an ECMO early-warning alert was triggered, on-call RRT members immediately responded and arrived on site within 10 min with standardized emergency materials. Based on clearly defined role responsibilities, two RRT physicians performed ultrasound-guided arteriovenous catheterization, while the nursing leader coordinated sterile field maintenance, medication administration, and resuscitation support. Concurrently, RRT nurses completed circuit priming, secured the extracorporeal circuit, and assisted physicians in bubble-free connection and ECMO initiation. After successful cannulation, a standardized transfer process was activated to ensure safe and seamless patient transport to the ICU for handover to MET members, with documentation completed jointly by both teams. During ECMO maintenance, MET members delivered refined management according to ECMO clinical standards, including environmental control, hemodynamic monitoring, coagulation assessment, sedation–analgesia management, and basic nursing care, forming a real-time response chain of “early warning–intervention–monitoring.” When criteria for ECMO weaning were met, MET members coordinated weaning procedures, monitored post-weaning vital signs, assessed vascular and tissue recovery via ultrasound, adjusted rehabilitation programs, and completed chain-management outcome records.

Prior to discharge or inter-departmental transfer, MET members provided structured medication guidance and health education. Follow-up was conducted at 1 week and 28 days after ECMO initiation to assess recovery progress and survival status, with all information systematically entered into the chain-management file. Finally, a feedback chain of “summary–evaluation–improvement” was applied to review the entire treatment course and continuously optimize workflow and team performance.

### 2.6 Evaluation indicators and methods of data collection

#### 2.6.1 Evaluation indicators

The primary evaluation indicators were the duration of the ECMO initiation stage (representing the time from the confirmation of ECMO treatment to the successful initiation of ECMO blood drainage), team preparation time (interval between the receipt of notification to confirm ECMO treatment to the complete assembly of personnel, supplies, and equipment), priming time (the time from the start of circuit priming by nurses to the successful completion of priming), the oxygenation index (P/F ratio) determined by blood gas analysis before ECMO initiation and 4 h after ECMO initiation (for VV-ECMO), and the lactic acid level associated with blood gas analysis measured before ECMO initiation and 4 h after ECMO initiation (for VA-ECMO).

The secondary evaluation indicators were the ECMO runtime (the time from the initiation of ECMO blood drainage and machine operation to ECMO weaning), rate of complications (the number of cases with complications during ECMO treatment/total number of cases receiving ECMO treatment), and patient outcomes (survival status at 28 days after ECMO initiation).

#### 2.6.2 Methods of data collection

For data on primary evaluation indicators, RRT members took on-site photographs at specific time points, namely, at the completion of team preparation, the start of priming, the completion of priming, and the initiation of ECMO blood drainage. The time of ECMO initiation, team preparation time, and priming time were calculated by extracting information on time differences between the photographs, which was then verified and collected by the data managers of the research team. Blood gas analysis was rechecked before ECMO initiation and 4 h after ECMO initiation, and the P/F ratio and lactic acid levels in the blood-gas reports were recorded.

For the secondary evaluation indicators, members of the research team collected disease-related information of the subjects from the electronic medical record system (EMRS).

### 2.7 Statistical analysis

Statistical analyses were performed using Stata 15 and IBM SPSS Statistics version 27. Data distributions were assessed using the Shapiro–Wilk normality test. Measurement data with normal distributions are expressed as mean ± standard deviation ( $\bar{x}\pm s$ ); independent-samples *t*-tests were used for intergroup comparisons, while paired-samples *t*-tests were used for intragroup comparisons. Non-normally distributed measurement data are expressed as median (interquartile range, IQR), with rank-sum tests used for intergroup comparisons and paired signed-rank tests for intragroup comparisons. Count data are presented as frequency and percentage (*n*, %), and differences between groups were compared using Pearson’s chi-square test or Fisher’s exact test. For categorical variables with small expected cell counts, Fisher’s exact test was used, and only *p*-values were reported.

To account for potential confounding from baseline imbalances and time-based allocation, multivariable linear regression was performed for continuous outcomes, including ECMO initiation time, preparation time, priming time, and ECMO runtime, adjusting for age, APACHE II score, ECMO type, and comorbidities. Multivariable logistic regression was used for binary outcomes, namely 28-day survival and complications, with adjusted odds ratios (ORs) and 95% confidence intervals (CIs) reported. Subgroup analyses by ECMO type (VV-ECMO vs. VA-ECMO) were also conducted.

## 3 Results

### 3.1 Comparison of general data between the two groups

The control group comprised 19 males and 9 females. The age was 58 58 (51.0, 68.5) years, with body mass index (BMI) values of 24.3 (21.4, 25.7) and APACHE II scores of 23.1 ± 8.4. Among them, 5 cases had no underlying diseases, while 23 cases had underlying diseases; 7 patients received venovenous ECMO (VV-ECMO) and 21 received veno-arterial ECMO (VA-ECMO). The experimental group included 20 males and 8 females, aged 53.5 (44.0, 59.5) years, with a BMI of 24.9 (22.6, 26.1) and APACHE II score of 26.1 ± 5.8. Among them, 5 cases had no underlying diseases and 23 cases had underlying diseases; 13 patients received VV-ECMO and 15 received VA-ECMO. No statistically significant differences were observed in the general data between the two groups (*p* > 0.05), as presented in Table 1.

**Table 1:** Comparison of general data between the two groups.

Variables	Control group	Experimental group	Statistical value	*p-*value
Number of cases (*n*)	28	28		
Sex (*n* [%])				
Female	9 (32.1)	8 (28.6)	0.08^*^	0.77
Male	19 (67.9)	20 (71.4)		
Age (years)	58 (51.0, 68.5)	53.5 (44.0, 59.5)	1.72^#^	0.09
BMI	24.3 (21.4, 25.7)	24.9 (22.6, 26.12)	−1.26^#^	0.21
APACHE II score (points)	23.1 ± 8.4	26.1 ± 5.8	−1.52	0.14
Underlying diseases [*n* (%)]				
None	5 (17.9)	5 (17.9)	0.00^*^	1.00
Yes	23 (82.1)	23 (82.1)		
ECMO mode [*n* (%)]				
Veno-venous (VV)	7 (25)	13 (46.4)		
Veno-arterial (VA)	21 (75)	15 (53.6)	2.80^*^	0.09

In the statistical values, # indicates the *Z*-value, * indicates the χ^2^ value, and the remainder are *t*-values.

### 3.2 Comparison of the durations of each link in the ECMO treatment process between the two groups

The times required for ECMO initiation, team preparation time, and priming time were significantly shorter in the experimental group, indicating improved process efficiency (all *p* < 0.05), as presented in Table 2.

**Table 2:** Durations of individual links in the ECMO treatment process in the two groups.

Group	Number of cases (*n*)	Team preparation time (min)	ECMO initiation time (min)	Priming time (min)
Control group	28	10 (8, 12)	69 (52.5, 87.5)	14.12 ± 2.83
Experimental group	28	6 (5, 7)	39 (35, 47.5)	11.29 ± 1.90
Statistical value		5.27^#^	4.98^#^	4.49
*p*-value		<0.001	<0.001	<0.001

In the statistical values, # indicates the *Z*-value, while the remainder are *t*-values.

### 3.3 Changes in oxygenation index and blood lactic acid level before and after ECMO initiation

Patients in both groups receiving VV-ECMO demonstrated significant improvements in oxygenation, with P/F ratios at 4 h after initiation markedly higher than baseline (both *p* < 0.05). Among patients receiving VA-ECMO, blood lactate (Lac) levels decreased at 4 h compared with pre-initiation values, although the reductions did not reach statistical significance (both *p* > 0.05). Moreover, no significant between-group differences were observed in the magnitude of P/F ratio improvement or lactate reduction at 4 h following ECMO initiation (both *p* > 0.05), as shown in Tables 3, 4.

**Table 3:** Comparison of changes in the oxygenation index (P/F ratio) before and after ECMO initiation in patients with VV-ECMO.

Group	Number of cases (*n*)	P/F ratio before ECMO (mmHg)	P/F ratio after ECMO (mmHg)	Difference in P/F ratio (before vs. after)	Statistical value (intragroup)	*p*-value (intragroup)
Control group	7	101.24 ± 43.13	189.78 ± 60.48	74.4 (69.4, 122.8)	−2.37^#^	0.02
Experimental group	13	93.8 ± 33.85	201.06 ± 74.86	107.26 ± 70.33	−5.50	0.001
Statistical value (intergroup)		0.43	−0.34	−0.66		
*p*-value (intergroup)		0.67	0.74	0.52		

In the statistical values, # indicates the *Z*-value, while the remainder are *t*-values.

**Table 4:** Comparison of changes in blood lactic acid (Lac) levels before and after ECMO initiation in patients with VA-ECMO.

Group	Number of cases (*n*)	Lac level before ECMO (mmol/L)	Lac level at 4 h after ECMO (mmol/L)	Difference in Lac level (before vs. after)	Statistical value (intragroup)	*p-*value (intragroup)
Control group	21	4.9 (2.8, 8.8)	4.4 (3.0, 8.5)	−0.1 (−1.6, 0.4)	0.92^#^	0.36
Experimental group	15	4.4 (2.9, 10.2)	4.3 (1.8, 11.4)	−0.3 (−2.1, 1.5)	0.34	0.74
Statistical value (intergroup)		−0.048^#^	0.64^#^	−0.40^#^		
*p*-value (intergroup)		0.96	0.52	0.69		

In the statistical values, # indicates the *Z*-value, while the remainder are *t*-values.

### 3.4 ECMO runtime, complication rates, and patient outcomes

Categorical variables were analyzed using Fisher’s exact test due to the small sample size, for which no test statistics are available. Among patients receiving VV-ECMO, the ECMO runtime in the experimental group was significantly shorter than that in the control group (*p* < 0.05). The rate of complications was lower in the experimental group than in the control group, and the 28-day survival rate was higher; however, no statistically significant differences were observed between groups in complication rates or 28-day survival outcomes (both *p* > 0.05). Among patients receiving VA-ECMO, the ECMO runtime in the experimental group was longer than that in the control group, while the rate of complications was lower and the 28-day survival rate was higher compared to the control group. However, none of these differences reached statistical significance (all *p* > 0.05), as presented in Tables 5, 6.

**Table 5:** ECMO runtime, complication rate, and outcomes of patients with VV-ECMO in both groups.

Group	Number of cases (*n*)	ECMO runtime (h)	Complications [*n* (%)]		28-day survival status after ECMO initiation [*n* (%)]	
			Yes	No	Survived	Deceased
Control group	7	229.5 (110.5, 335)	3 (42.9)	4 (57.2)	3 (42.9)	4 (57.1)
Experimental group	13	105 (51.5, 161)	2 (15.4)	11 (84.6)	7 (53.8)	6 (46.2)
Statistical value		2.02^#^	--		--	
*p*-value		0.04	0.29^*^		1.00^*^	

In the statistical values, # indicates the *Z*-value; * indicates Fisher’s exact test.

**Table 6:** ECMO runtime, complication rate, and outcomes of patients with VA-ECMO in both groups.

Group	Number of cases (*n*)	ECMO runtime (h)	Complications [*n* (%)]		28-day survival status after ECMO initiation [*n* (%)]	
			Yes	No	Survived	Deceased
Control group	21	51.5 (22, 186)	6 (28.6)	15 (71.4)	7 (33.3)	14 (66.7)
Experimental group	15	91 (40.5, 239)	1 (6.7)	14 (93.3)	8 (53.3)	7 (46.7)
Statistical value		0.289^#^	--		--	
*p*-value		0.77	0.20^*^		0.31^*^	

In the statistical values, # indicates the *Z*-value; * indicates Fisher’s exact test.

### 3.5 Adjusted analyses

As shown in Table 7, after adjustment, the experimental group still had significantly shorter ECMO initiation time (*B* = −23.83, 95% CI -34.05 to −13.61, *p* < 0.001), time preparation time (*B* = −3.57, 95% CI -4.80 to −2.34, *p* < 0.001), and priming time (*B* = −2.80, 95% CI -4.25 to −1.35, *p* < 0.001) compared with the control group.

**Table 7:** Multivariable regression analyses.

Outcome	Adjusted effect (95% CI)	*p*-value
ECMO initiation time (min)	aMD = −23.83 (−34.05, −13.61)	<0.001
Team preparation time (min)	aMD = −3.57 (−4.80, −2.34)	<0.001
Priming time (min)	aMD = −2.80 (−4.25, −1.35)	<0.001
VV-ECMO runtime (h)	aMD = −105.64 (−284.37, 73.10)	0.23
VA-ECMO runtime (h)	aMD = +1.87 (−65.48, 69.21)	0.96
28-day survival (alive vs. dead)	aOR = 0.70 (0.21, 2.35)	0.56
Complication (yes vs. no)	aOR = 0.43 (0.11, 1.71)	0.23

aMD, adjusted mean difference, derived from linear regression (unstandardized B); aOR, adjusted odds ratio, derived from logistic regression (Exp[B]); All models were adjusted for age, APACHE II score, ECMO type (VV or VA), and presence of comorbidities; VV-ECMO and VA-ECMO runtimes were analyzed separately using stratified linear regression (without adjusting for ECMO type); 28-day survival and complication outcomes were analyzed using binary logistic regression.

No statistically significant differences were observed between groups for ECMO runtime (VV-ECMO: *p* = 0.23; VA-ECMO: *p* = 0.96), 28-day survival (OR = 0.70, 95% CI 0.21–2.35, *p* = 0.56), or complication rates (OR = 0.43, 95% CI 0.35–6.77, *p* = 0.57).

## 4 Discussion

### 4.1 Reliability and safety of the dual-team collaboration model guided by chain management in ECMO emergency care

This study demonstrated that the dual-team collaboration model guided by chain management provides reliable and safe support for ECMO initiation in critically ill ICU patients. Several factors may explain these findings. First, the development of the model was grounded in a systematic review of the literature and informed by authoritative domestic and international guidelines and expert consensus on ECMO emergency management, ensuring a strong theoretical and evidence-based foundation for the protocol. Second, multidisciplinary expert consultations—covering critical care, emergency medicine, and cardiovascular surgery-facilitated refinement of the workflow to better align with clinical realities, thereby enhancing the model’s feasibility and rigor. In addition, all members of the RRT-MET team completed standardized ECMO-specific training and certification, which improved the consistency and safety of technical operations. The inclusion of daily on-call ECMO RRT personnel further ensured rapid response capability. Prior to implementation, a pilot test was conducted, demonstrating that the model could effectively streamline team coordination and resource allocation, shortening the interval from RRT activation to ECMO initiation from approximately 70 min to 40 min. This improvement reflects enhanced process integration and operational efficiency. Overall, the findings suggest that the chain management–guided dual-team collaboration model strengthens the continuity, timeliness, and standardization of ECMO emergency care.

### 4.2 The RRT-MET collaborative model based on chain management achieves high efficiency and Favorable outcomes in ECMO emergency care for ICU patients

This study indicates that the dual-team collaboration model guided by chain management enhances the efficiency of ECMO emergency care and contributes to favorable clinical process metrics in ICU patients. By standardizing the ECMO initiation workflow and enabling rapid assessment and intervention upon deterioration, the model significantly shortened preparation time, priming time, and ECMO initiation time (all *p* < 0.05). These findings align with those of Burrell et al. (17), who reported that dedicated mobile ECMO teams markedly reduce the time to ECMO initiation. Although previous evidence has shown that RRTs can reduce cardiac arrest and in-hospital mortality, their effectiveness depends on standardized organizational structures and procedures (18, 19). Chain management provides a time-bound, sequence-driven framework that requires personnel to complete designated tasks within specific time windows, thereby reducing interdepartmental coordination delays and achieving seamless operational integration (20). In this study, the integration of chain management into the ECMO emergency care system—via the feed-forward, in-process, and feedback chains—was a central mechanism for improving workflow efficiency. This full-cycle approach included regular team training before ECMO initiation, rapid RRT response and standardized catheterization with simultaneous circuit priming during initiation, structured transfer to MET members for continuous management, and post-decannulation review and optimization through chain-management documentation.

Consistent with expected physiological effects, patients in both groups demonstrated significant improvement in oxygenation (P/F ratios) at 4 h after VV-ECMO initiation (*p* < 0.05). In VA-ECMO patients, lactate levels showed a decreasing trend in both groups, although the differences were not statistically significant, possibly due to the limited sample size or short observation window. However, while physiological indicators (e.g., oxygenation and lactate levels) improved after ECMO initiation in both groups, no significant between-group differences were observed, further indicating that the benefits of the model may be primarily reflected in process optimization rather than immediate clinical outcomes. This observation is broadly consistent with previous studies (21), which suggest that standardized, multidisciplinary ECMO collaboration programs may contribute to improved outcomes; however, such benefits are not always detectable in early or small-sample studies.

### 4.3 RRT-MET collaborative model based on chain management may have the potential to improve the prognosis of ICU patients undergoing ECMO

ECMO is a highly resource-intensive and invasive life-support modality typically applied to critically ill patients, and its clinical use is accompanied by substantial risks and complex management challenges. ECMO-supported transport represents the most advanced form of critical care transfer, further underscoring the inherent danger and technical difficulty of ECMO management (22, 23). Despite progressive refinement in ECMO technology and clinical experience, the mortality rate among ECMO-treated patients remains high. According to recent ELSO data, the overall survival rate for adults receiving ECMO is approximately 51% (24), consistent with global reports (25).

In the present study, several findings suggest potential trends toward improved clinical outcomes with the chain management–guided RRT–MET collaborative model. Among VV-ECMO recipients, the ECMO runtime was significantly shorter in the experimental group than in the control group (*p* < 0.05). This pattern aligns with the more pronounced improvement in oxygenation (P/F ratio) observed in the experimental group, indicating that the standardized, time-critical workflow facilitated by chain management may accelerate physiological recovery and shorten dependence on ECMO support. However, this association was not statistically significant after adjustment, likely due to the limited sample size (*n* = 20 VV-ECMO patients), which reduced statistical power. For VA-ECMO patients, ECMO runtime was longer in the experimental group, although the difference was not statistically significant (*p* > 0.05). This trend may reflect clinical variability and the need for prolonged support in more severe cases rather than a direct effect of the intervention. Previous studies have suggested that appropriately prolonged ECMO support may facilitate organ recovery in selected patients (26, 27); however, such interpretations cannot be confirmed within the present study design. Regarding safety outcomes, the experimental group showed numerically lower complication rates under both VV- and VA-ECMO support, although the differences were not statistically significant (all *p* > 0.05). While these trends may be associated with improved workflow standardization, clearer role delineation, and enhanced team coordination, the absence of statistical significance limits definitive conclusions.

Similarly, survival analyses demonstrated favorable but non-significant trends, with higher 28-day survival rates observed in the experimental group in both VV-ECMO (53.8% vs. 42.9%) and VA-ECMO subgroups (53.3% vs. 33.3%). These findings should be interpreted with caution, as the study was not powered to detect differences in survival outcomes. Although previous studies have reported that multidisciplinary ECMO management protocols may improve patient outcomes (28), such benefits were not conclusively demonstrated in the current study. Several factors may explain the lack of statistically significant differences in clinical outcomes, including the small sample size, subgroup heterogeneity, and variability in underlying disease severity and weaning strategies. Future studies should incorporate larger, multicenter samples, stratified analyses, and standardized clinical protocols to better evaluate the prognostic impact of this model. Overall, the primary strength of this study lies in demonstrating improved process efficiency in ECMO initiation and management. While trends toward improved clinical outcomes were observed, these findings remain exploratory and should not be interpreted as evidence of definitive clinical benefit.

### 4.4 Implications for clinical practice

Implementing a chain management–based RRT–MET collaborative model may provide meaningful clinical value for ECMO care in ICUs. This structured approach strengthens continuity and timeliness of decision-making, facilitates earlier activation of ECMO pathways, and standardizes peri-ECMO management through seamless multidisciplinary coordination. The reduced VV-ECMO duration suggests improved treatment efficiency and potentially accelerated pulmonary recovery. Although benefits in VA-ECMO were less pronounced, favorable trends indicate potential contributions to cardiopulmonary stabilization. The lower incidence of complications and higher 28-day survival rates further highlight the model may have the potential to enhance patient safety and prognosis by reducing process-related risks through standardized workflows and strengthened communication.

While the implementation of a dual-team model may introduce additional organizational and training costs, these investments could be offset by gains in operational efficiency and potential contributions to improved clinical outcomes. In particular, observed reductions in ECMO initiation time and VV-ECMO duration suggest more efficient resource utilization, potentially decreasing overall ICU length of stay and ECMO-related resource consumption. Improved coordination and standardized workflows may also reduce process-related complications, thereby potentially lowering downstream treatment costs. From a value-based care perspective, this model emphasizes optimizing both efficiency and patient outcomes, aligning with the goal of maximizing clinical benefit relative to resource use. However, formal cost-effectiveness analyses were not performed in this study; future research should incorporate economic evaluations to quantify the financial implications of this collaborative model.

### 4.5 Strengths and limitations

This study is among the first to systematically evaluate a chain management–based RRT–MET collaborative model in ECMO care, an area where standardized operational frameworks remain limited. By comparing multiple clinical outcomes between groups, the findings provide preliminary evidence supporting the potential value of structured multidisciplinary coordination. The inclusion of both VV-ECMO and VA-ECMO patients and the assessment of multidimensional outcomes further strengthen the comprehensiveness of the evaluation.

This study has several limitations. First, the non-concurrent design (control: 2023; experimental: 2024–2025) may introduce temporal bias from potential improvements in team experience, institutional resources, or protocol changes. Time-related process indicators were partially measured using photographic time points, which could be prone to measurement bias, although standardized training, consistent equipment, and blinded outcome assessment were applied to mitigate this. Second, as a single-center, non-randomized study with a modest sample size (*n* = 56), statistical power was limited, particularly for binary outcomes (e.g., survival, complications) and subgroup analyses. Clinically relevant baseline imbalances, higher VV-ECMO proportion and APACHE II scores in the experimental group, may have influenced outcomes, and despite multivariable adjustment, residual confounding cannot be excluded; notably, the direction of the 28-day survival estimate reversed after adjustment (aOR = 0.70, *p* = 0.56), suggesting observed benefits may partly reflect these imbalances. Third, clinical heterogeneity and variations in ECMO decision-making could have introduced additional confounding, and no formal cost-effectiveness analysis was performed. Finally, the model was implemented in a tertiary ECMO-capable ICU, limiting generalizability to non-ECMO centers, and patient outcomes were assessed only up to 28 days post-ECMO initiation; longer-term survival, including 3-month and 1-year outcomes, was not evaluated. Future multicenter studies with larger sample sizes, inclusion of both ECMO and non-ECMO centers, rigorous baseline control, and extended follow-up are warranted to validate these findings, clarify the long-term impact, and determine broader applicability of this collaborative model.

## 5 Conclusion

In summary, the chain management–based RRT–MET collaborative model significantly improved ECMO initiation efficiency, including reductions in team preparation, priming, and initiation times in ICU patients. However, no statistically significant improvements were observed in complication rates, 28-day survival, or VV-ECMO runtime. Although favorable trends in safety and survival outcomes were noted, these findings remain inconclusive and should be interpreted with caution. This study provides an initial framework for integrating RRT and MET within a chain management–guided, full-cycle ECMO care pathway, highlighting its value in process optimization rather than confirmed clinical benefit. Future multicenter, large-sample, randomized controlled studies are required to determine whether improvements in efficiency can translate into meaningful improvements in patient outcomes.

## Data Availability

The raw data supporting the conclusions of this article will be made available by the authors, without undue reservation.
